# Harmine inhibits breast cancer cell migration and invasion by inducing the degradation of Twist1

**DOI:** 10.1371/journal.pone.0247652

**Published:** 2021-02-24

**Authors:** Ebtesam Nafie, Jade Lolarga, Brandon Lam, Jonathan Guo, Elnaz Abdollahzadeh, Sandy Rodriguez, Carlotta Glackin, Junjun Liu

**Affiliations:** 1 Beckman Research Institute, City of Hope, Duarte, CA, United States of America; 2 Zoology Department, Faculty of Sciences, Benha University, Benha, Egypt; 3 Department of Biological Sciences, California State Polytechnic University, Pomona, CA, United States of America; 4 Department of Biological Sciences, California State University, Long Beach, CA, United States of America; University of Alabama at Birmingham, UNITED STATES

## Abstract

Breast cancer is the leading cause of cancer-related deaths in the United States. The majority of deaths (90%) in breast cancer patients is caused by invasion and metastasis–two features related to the epithelial-to-mesenchymal transition (EMT). Twist1 is a key transcription factor that promotes the EMT, which leads to cell migration, invasion, cancer metastasis, and therapeutic resistance. Harmine is a beta-carboline alkaloid found in a variety of plants and was recently shown to be able to induce degradation of Twist Family BHLH Transcription Factor 1 (Twist1) in non-small cell lung cancer cells (NSCLC). In this study, we show that harmine can inhibit migration and invasion of both human and mouse breast cancer cells in a dose-dependent manner. Further study shows that this inhibition is most likely achieved by inducing a proteasome-dependent Twist1 degradation. At the concentrations tested, harmine did not affect the viability of cells significantly, suggesting that its inhibition of cancer cell migration and invasion is largely independent of its cytotoxicity, but due to its ability to affect regulators of EMT such as Twist1. This result may facilitate the development of strategies that target Twist1 to treat metastatic breast cancer, as Twist1 is expressed at a high level in metastatic breast cancer cells but not in normal cells.

## Introduction

In 2020, an estimated 276,480 new cases of invasive breast cancer are expected to be diagnosed in women in the U.S., along with 48,530 new cases of non-invasive (in situ) breast cancer [1]. The majority of deaths from breast cancer are not due to the primary tumor itself, but are the result of metastasis to other parts of the body [2]. Metastasis is a process comprised of a series of sequential steps, starting with local invasion of surrounding tissues by cells originating from the primary tumor and continuing until tumor cells invade and intravasate into blood or lymphatic vessels [3, 4].

At the initial stage of metastasis, invasive tumor cells first alter cell-to-cell adhesion and cell adhesion to the extracellular matrix (ECM). Proteins in the Cadherin family are important in mediating cell-to-cell adhesion and play a predominant role in breast cancer metastasis [5]. E-Cadherin maintains cell-cell junctions and its down-regulation correlates with the development of metastatic breast cancer cells [6]. On the other hand, N-Cadherin is closely associated with mesenchymal cells and related to epithelial-to-mesenchymal transition (EMT) during the gastrulation stage [7]. Increasing evidence shows that EMT is associated with cancer progression [8, 9] by assisting invasion and intravasation into the bloodstream and inducing proteases involved in the degradation of the ECM [10]. During EMT, cells undergo changes from an epithelial phenotype to a mesenchymal-like phenotype [11]. EMT starts with the disintegration of cell-cell adhesion by loss of epithelial markers, such as E-Cadherin, and the expression of mesenchymal markers, such as Vimentin and N-Cadherin. Accordingly, the expression of transcriptional repressors of E-Cadherin, including zinc finger E-box-binding homeobox 1 (ZEB1), zinc finger E-box-binding homeobox 2 (ZEB2), twist-related protein (Twist), zinc finger protein, Snail, and Slug, is associated with poor prognosis in breast carcinoma [12].

The Twist family of basic helix-loop-helix transcription factors, which includes Paraxis, Scleraxis, Hand1, Hand2, Twist1, and Twist2 [13], is involved in EMT [14]. Both Twist1 and Twist2 function in the transcriptional regulation of developmental processes, but Twist1 is better studied and is a known activator of EMT in cancer cells [15]. It promotes EMT by activating several target genes that promote cellular de-differentiation and cell mobility. In addition, Twist1 is also reported to promote the cancer stem cell phenotype, inhibit apoptosis, and contribute to chemotherapy resistance [16]. Overexpression of Twist1 is common in metastatic carcinomas including in aggressive and metastatic forms of breast cancer [16–18].

As a master regulator of EMT in breast epithelial cells, Twist1 is a promising target for metastatic breast cancer therapy. What makes Twist1 a particularly attractive target is that it is rarely expressed in normal adult tissues [19]. This makes it relatively safe to target Twist1 in the treatment of cancers such as metastatic breast cancer. Successful inactivation of Twist1 in cancer cells by siRNA or chemotherapeutic approaches has been reported [20–23], and inhibitors targeting either the upstream regulator or downstream effector of Twist1 signaling have also been identified for cancer therapy [24].

Harmine is a beta-carboline alkaloid found in a variety of plants, such as the Middle Eastern plant harmal or Syrian rue (Peganum harmala) and the South American vine. It is reported to have cytotoxic activity against human tumor cell lines [25]. In MDA-MB-231 breast cancer cells that overexpress breast cancer resistance protein (BCRP), harmine inhibits BCRP [26]. Harmine is also reported to antagonize transcriptional coactivator with PDZ-binding motif (TAZ), suppress breast cancer cell proliferation and migration, and promote cancer cell apoptosis in vitro [27]. Recently, in an unbiased screen, harmine was identified as the first pharmacologic inhibitor of Twist1 with significant anti-tumor activity in oncogene-driven lung cancer [28]. Treatment with harmine causes Twist1 degradation and induces senescence or apoptosis in NSCLC cells [28].

Given the importance of Twist1 in promoting EMT in breast carcinoma cells, in this study, we investigate the effect of harmine treatment in both human and mouse breast cancer cells and find that in both cases, harmine induces a proteasome-mediated Twist1 degradation and inhibits cancer cell invasion.

## Materials and methods

### Cell lines

As previously described [29], BT549 (RRID:CVCL_1092) Twist- cells were generated by transfecting BT549 cells (ATCC HTB-122) purchased from ATCC with short hairpin RNA (shRNA) that knocks down the expression of endogenous Twist1; whereas BT549 Twist+ cells stably express control scrambled shRNA and maintain high level expression of endogenous Twist1. Mouse 4T1 (RRID: CVCL_0125) cells were provided by Dr. Jing Yang at the University of California San Diego and cultured as described [20]. 4T1 Twist- cells stably express siRNA that knocks down the expression of endogenous Twist1, and 4T1 Twist+ stably express control siRNA that does not affect the expression of endogenous Twist1. Unless otherwise indicated, BT549 cells were cultured in RPMI-1640 medium containing 2 mM L-glutamine and10 mM HEPES, and supplemented with 10% Fetal Bovine Serum (FBS) and 0.023 IU/ml insulin; the 4T1 cells were cultured in RPMI-1640 medium containing 2 mM L-glutamine, 10 mM HEPES and supplemented with 10% FBS. All human cell lines (BT549 Twist- and Twist+) have been authenticated using STR profiling within the last three years. All experiments were performed with mycoplasma-free cells.

### Wound healing assay

Cell migration was measured using a wound healing assay. BT549 and 4T1 cells were cultured in 24-well plates with appropriate media (see Cell Lines). Once cells formed a confluent monolayer, the cells were pretreated with 10 μg/ml mitomycin c (Research Products International Corp) in serum-free media at 37°C in 5% CO_2_ incubtor for 2 hrs. After washing with 1 x PBS, a sterile pipette tip was used to inflict a ~1.5 mm wide ‘wound’. The PBS was then replaced with cell culture media supplemented with 2.5% FBS. Cells were left either untreated or treated with harmine at 5, 10, or 20 μM for BT549 cells, or 60, 120, or 180 μM for 4T1 cells, due to their higher resistance to harmine treatment. Images of the wound margins were captured using an inverted light microscope (Leica DM4000, Leica Biosystems, Germany) at 0, 24, 48, and 72 hr for BT549 cells, and 0, 6, and 12 hr for 4T1 cells. The wound healing rate was calculated using the following formula: (average wound margin in mm at 0 hr ‑ average wound margin at 24 hr) / average wound margin at 0 hr.

### 3D invasion assay

Cells were diluted to 500–1,000 cells/μL in media and pipetted onto the inner lid of a 10 cm cell culture dish containing 5 mL of sterile PBS at 20 μL/droplet. The lid was placed on the culture dish which was incubated in a 5% CO_2_ incubator at 37°C for 48–72 hrs until cell spheroids formed. Cell spheroids were collected and allowed to settle at the bottom of a microcentrifuge tube for 10 minutes. Matrigel (Corning) and Type I collagen (Corning) were gently mixed at a 1:1 ratio with 40 μL of cell spheroids and then placed in a well in a 24-well plate, which was covered with 1 mL of pre-warmed complete cell culture medium with 2.5% FBS. For harmine treatment, either DMSO or harmine was added to each well to a final concentration of 5 μM, 10 μM, or 20 μM for BT549 Twist+ cells, or 60 μM, 120 μM, or 180 μM for 4T1 Twist+ cells. The plate was incubated in 5% CO_2_ incubator at 37°C and the invasion results were recorded at 0, 24, and 48 hrs. The invasion areas were measured with ImageJ.

### CCK8 assay

Cells were plated in 96 well plates at a density of 5x10^3^ cells per well. Following 24 hr of incubation, cells were either left untreated or treated with dimethyl sulfoxide (DMSO) or harmine (Indofine Chemical Company, Cat# 442-51-3, Lot# 1505603) at concentrations of 20 or 40 μM for 48 hr for BT549 cells, or 60, 120, or 180 μM for 48 hr for 4T1 cells, followed by addition of 10 μl CCK8 reagent to each well. Absorbance was measured at 450 nm using a plate reader. Optical density (OD) was calculated for cell viability assays. Cell viability (%) = OD (treated) / OD (control) x100%.

### Western blotting

Cells were plated in a 100 mm culture plate with 10 mL of media. Harvested cells were washed with 1 x phosphate buffered saline (PBS) and lysed with 1 x RIPA buffer (Thermo Scientific, Waltham, MA, USA, Cat # 89900). For Western blotting analysis, 20 μg of protein was resolved with 10% sodium dodecyl sulfate polyacrylamide gel electrophoresis (SDS-PAGE) resolving gel and transferred to nitrocellulose membranes (GE Healthcare Life science, Pittsburgh, PA, USA). Membranes were probed with anti-Twist1 monoclonal antibody (Twis2C1a, sc-81417, Santa Cruz Biotechnology, Santa Cruz, CA, USA) at a dilution of 1:250, anti-beta actin monoclonal antibody (Proteintech, Rosemont, IL, USA, Cat # 66009–1,) at a dilution of 1:5,000, and anti-GAPDH monoclonal antibody (Proteintech, Rosemont, IL, USA, Cat # 60004–1,) at a dilution of 1:10,000. The secondary antibodies used were horseradish peroxidase (HRP)-goat anti-mouse IgG (H+L) polyclonal antibody (Invitrogen, Carlsbad, CA, USA, Cat # 31430,). Images were detected using an ECL chemiluminescent substrate (Thermo Scientific, Waltham, MA, USA, Cat # 34095,) and analyzed using a ChemiDoc imager (Bio-Rad Laboratories, Hercules, CA, USA).

### Reverse transcription quantitative PCR (RT-qPCR)

After harmine treatment, cells were harvested and total RNA was extracted using a RNeasy Plus Mini Kit (Qiagen, Hilden, Germany) according to the manufacturer’s instructions. RNA quality was evaluated using a 1% formaldehyde-agarose gel. In order to prevent RNA secondary structures, total RNA was denatured before running on a gel. To remove genomic DNA contaminants, total RNA extracts were treated with RQ1 RNase-Free DNase (Promega, Madison, WI, USA). For mRNA analysis, equal volumes of total RNA were used for cDNA synthesis using SuperScript II Reverse Transcriptase (Thermo Fisher Scientific, Waltham, MA, USA) according to the manufacturer’s instruction. RT-qPCR was performed using Maxima SYBR Green/ROX qPCR Master Mix (2x) (Thermo Fisher Scientific, Waltham, MA, USA) with equal volumes of cDNA and specific primers on a CFX96 Touch Real-Time PCR Instrument (Bio-Rad Laboratories, Hercules, CA, USA) with the following settings: 1 x 95°C 10 minutes; 40 x (95°C 15 seconds, 60°C 30 seconds, 72°C 30 seconds); followed by melt curve analysis. Twist1 expression levels were analyzed by calculating ddC_t_ and using the 2^-ddCt^ method; β-actin, glyceraldehyde 3-phosphate dehydrogenase (GAPDH), and Tubulin α 1a were used for normalization. Primer sequences are listed below from 5’ to 3’:

Human ACTB_F CACCATTGGCAATGAGCGGTTC
Human ACTB_R AGGTCTTTGCGGATGTCCACGT
Human GAPDH_F TGCACCACCAACTGCTTAGC
Human GAPDH_R GGCATGGACTGTGGTCATGAG
Human TUBA1A_F CGGGCAGTGTTTGTAGACTTGG
Human TUBA1A_R CTCCTTGCCAATGGTGTAGTGC
Human TWIST1_F GCCAGGTACATCGACTTCCTCT
Human TWIST1_R TCCATCCTCCAGACCGAGAAGG
Mouse TWIST1_F CGGGTCATGGCTAACGTG
Mouse TWIST1_R CAGCTTGCCATCTTGGAGTC
Mouse Snail_F CCCTCATCTGGGACTCTCTCC
Mouse Snail_R CCAGGCTGAGGTACTCCTTGT
Mouse BMI1_F AATTAGTCCCAGGGCTTTTCAA
Mouse BMI1_R TCTTCTCCTCATCTGCAACTTCTC
Mouse ZEB2_F GAGCTTGACCACCGACTC
Mouse ZEB2_R TTGCAGGACTGCCTTGAT
Mouse ACTIN_F CTGTCCCTGTATGCCTCTG
Mouse ACTIN_R ATGTCACGCACGATTTCC
Mouse GAPDH_F GTTGTCTCCTGCGACTTCA
Mouse GAPDH_R GGTGGTCCAGGGTTTCTTA
Mouse E-Cadherin_F AGACTCCCGATTCAAAGTGG
Mouse E-Cadherin_R TTCAGCGTCACTTTGGTAGACA
Mouse N-Cadherin_F CTGCCACTTTTCCTGGGTTTC
Mouse N-Cadherin_R ACGGTGTATGCTGTGAGAAGC
Mouse Vimentin_F CCCTCACCTGTGAAGTGGAT
Mouse Vimentin_R TCCAGCAGCTTCCTGTAGGT

### Statistical analysis

All data were analyzed using Prism 9 (GraphPad Software). All qPCR data were analyzed using multiple t-test, one unpaired t test per row. Statistical Significance method: correct for multiple using the Holm-Sidak method, definitions of statistical significance are * p < .05, ** p < .01, *** p < .001, **** p < .0001.

## Results

### Harmine inhibits breast cancer cell migration and invasion

**Fig 1:**
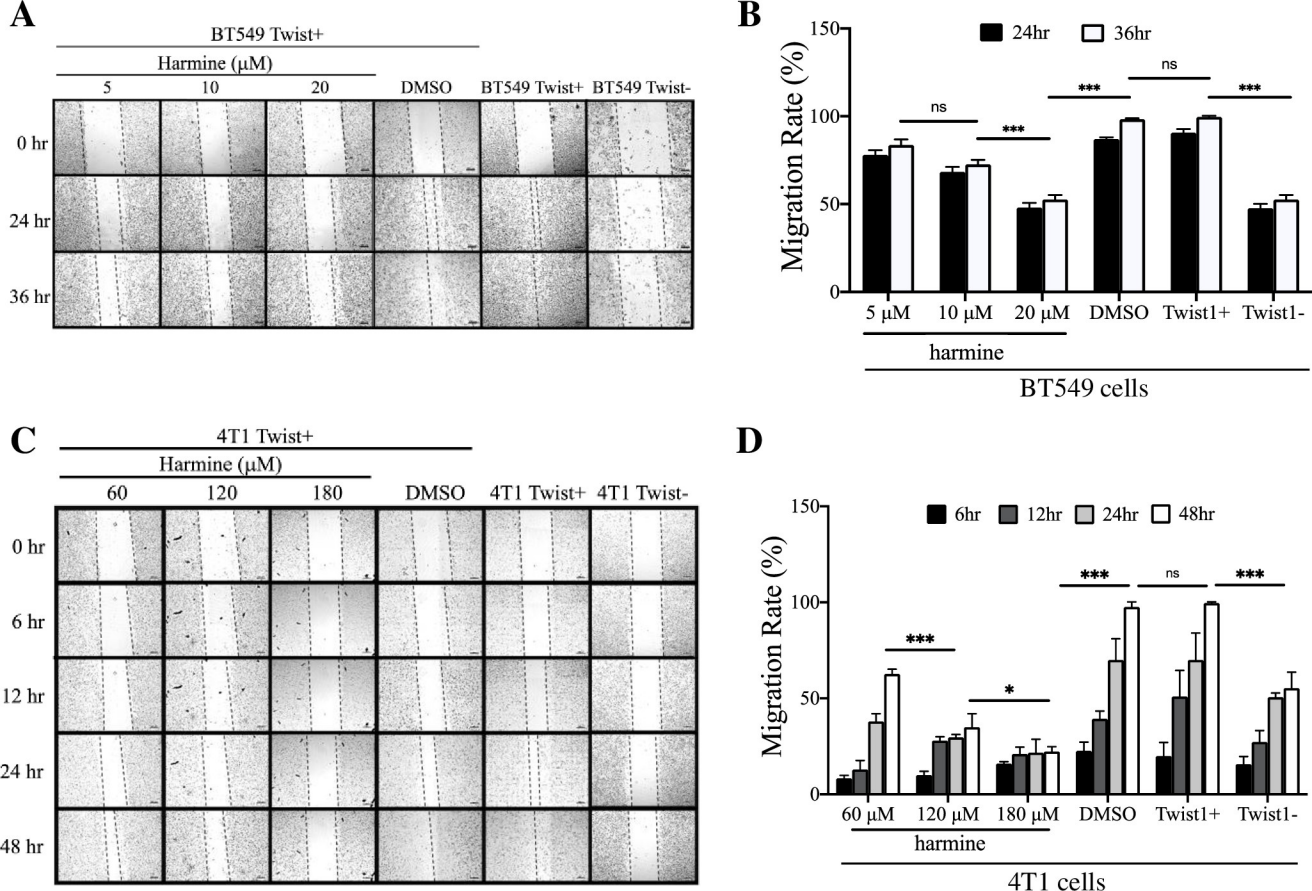
Harmine inhibits migration of breast cancer cells in wound healing assays. A, BT549 cells were treated with harmine (5, 10, 20 μM) or DMSO (solvent control) and images were captured at different time points (0, 24, and 36 hr). Untreated BT549 Twist+ and BT549 Twist- cells were included as positive and negative controls for migration, respectively. Images are from a single experiment. The scale bars represent 200 μm. B, Quantitative analysis of results from three independent experiments including the one shown in A. Statistical significance was determined based on data from 36 hr. C, 4T1 cells were treated with harmine (60, 120, 180 μM) or DMSO (solvent control) and images were captured at different time points (0, 6, 12, 24, and 48 hr). Untreated 4T1 Twist+ and 4T1 Twist- cells were included as positive and negative controls for migration, respectively. The black particles in images under 120 μM and 180 μM are harmine. Images are from a single experiment. The scale bars represent 200 μm. D, quantitative analysis of results from three independent experiments including the one shown in C. Statistical significance was determined based on the data from 48 hr. Definitions of statistical significance are * p < .05, ** p < .01, *** p < .001, and **** p < .0001.

Harmine causes NSCLC cell senescence and apoptosis by inducing Twist1 degradation. Since Twist1 is a well-documented regulator of breast cancer metastasis, we first wanted to find out whether harmine could inhibit cancer cell migration and invasion, the initial step towards metastasis. For this purpose, we first performed a wound healing assay with BT549 Twist+ cells to determine the effect of harmine on cell migration. S1A Fig shows that the Twist1 transcript level in BT549 Twist+ cells remains high with the transfection of scrambled shRNA; whereas in BT549 Twist- cells, Twist1 expression has been reduced significantly by stable transfection of shRNA targeting Twist1. There was no difference in migration between untreated BT549 Twist+ cell and the cells treated with DMSO. We found that at 24 hr, there appeared to be a dose-dependent inhibition of cell migration, but at 36 hr after the wound generation, cells treated with DMSO closed the gap completely, while the gaps in cells treated with harmine at 5, 10, and 20 μM remained (Fig 1A). Untreated BT549 Twist+ and BT549 Twist- cells were included as migration controls. As expected, BT549 Twist+ cells closed the gap at 36 hr, whereas the gap in BT549 Twist- cells remained after a 36 hr incubation, a result similar to that of BT549 Twist+ cells treated with 20 μM harmine. This result suggests that harmine inhibits migration of BT549 Twist+ cells, and that inhibition appears to be dose dependent (Fig 1B).

To find out whether harmine also inhibits migration of other types of breast cancer cell, we performed the same assay with mouse 4T1 cells. As in BT549 cells, the Twist1 transcript level in 4T1 Twist+ cells remains high; whereas in 4T1 Twist- cells, Twist1 expression is reduced significantly by siRNA targeting Twist1 (S1B Fig). Unlike in the assay with BT549 cells, we found that a significantly higher concentration (120 μM) of harmine is needed to effectively inhibit cell migration, most probably due to the fact that compared to BT549 cells, 4T1 Twist+ cells express a higher level of Twist1 and are highly invasive. There was no significant difference in migration between untreated 4T1 Twist+ cells and 4T1 Twist+ cells treated with DMSO (Fig 1C). The untreated 4T1 Twist+ cells and 4T1 Twist+ cells treated with DMSO closed the gap completely at 48 hr post wound; whereas cells treated with harmine at concentrations of 60 μM, 120 μM, and 180 μM did not. Untreated 4T1 Twist+ and Twist- cells were included as positive and negative controls, respectively. As expected, there is a significant difference in migration between them. We noticed that with an increase in harmine concentration from 60 μM to 180 μM, inhibition of migration was significantly enhanced (Fig 1D). These results further support our observation that harmine inhibits migration of breast cancer cells in a dose-dependent manner.

**Fig 2:**
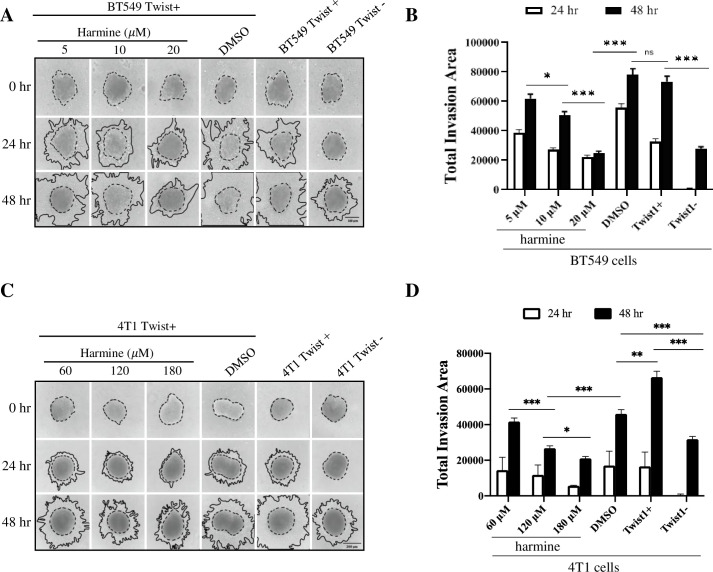
Harmine inhibits the invasion of breast cancer cells. The invasiveness of breast cancer cells was studied in a 3D setting (see Materials and Methods). A, BT549 cells were treated with harmine (5, 10, 20 μM) or DMSO (solvent control) and images were captured at different time points (0, 24 and 48 hr). Untreated BT549 Twist+ and BT549 Twist- cells were included as positive and negative controls, respectively. Images are from a single experiment. B, quantitative analysis of results from three independent experiments including the one shown in A. Statistical significance was determined based on data from 48 hr. C, 4T1 cells were treated with harmine (60, 120, 180 μM) or DMSO (solvent control) and images were captured at different time points (0, 24 and 48 hr). Untreated 4T1 Twist+ and 4T1 Twist- cells were included as positive and negative controls, respectively. Images are from a single experiment. D, quantitative analysis of results from three independent experiments including the one shown in C. Statistical significance was determined based on the data from 48 hr. Definitions of statistical significance are * p < .05, ** p < .01, *** p < .001, and **** p < .0001.

To understand the effect of harmine on breast cancer cell invasion, we performed invasion assays in a 3D setting (see Methods and Materials) with both BT549 cells and 4T1 cells. We first treated cells with either DMSO or harmine at various concentrations, then let them grow into spheroids that were subsequently implanted in a 3D matrix. Cell invasion, represented by expansion of the spheroids, was recorded at 24 hr and 48 hr after implantation, and the areas of expansion were analyzed by ImageJ. In BT549 cells, there was no significant difference in invasiveness between untreated BT549 Twist+ cells and BT549 Twist+ cells treated with DMSO. Inhibition of invasion appeared to be dose-dependent. After treatment with 20 μM harmine, expansion of the spheroids was reduced to a level similar to that of BT549 Twist- cells (Fig 2A and 2B). Similar results were obtained with 4T1 cells. With harmine treatment at concentrations of 60, 120, and 180 μM, cell invasion was reduced in a dose dependent manner (Fig 2C and 2D). At concentrations of 120 and 180 μM, cell invasion was reduced to a level similar to that in 4T1 Twist- cells. We noticed that compared to the invasiveness of untreated 4T1 Twist+ cells, DMSO causes significant inhibition of invasiveness, but the difference in invasiveness between cells treated with DMSO and cells treated with 120 or 180 μM harmine was more significant, suggesting that harmine indeed inhibits cellular invasion.

In summary, the results described above demonstrate that harmine inhibits the migration and invasion of breast cancer cells in a dose dependent manner.

### Harmine does not affect cell viability with significant difference at the concentrations tested

**Fig 3:**
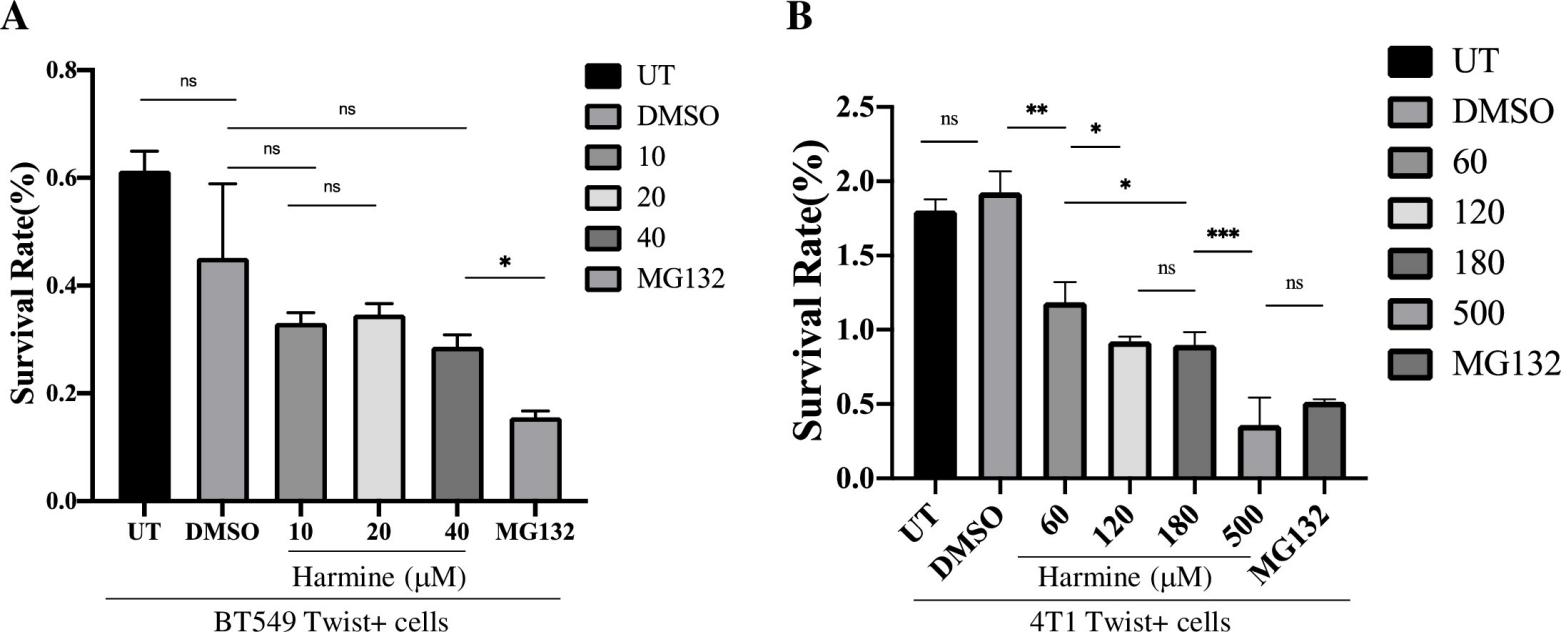
Harmine does not affect the viability BT549 cells, but inhibits the proliferation of 4T1 cells to some extent at the concentrations used in migration and invasion assays. The viabilities of breast cancer cells treated with harmine at concentrations corresponding to those used in wound healing and invasion assays were analyzed (see Materials and Methods). A, Harmine does not affect the viability of BT549 Twist+ cells significantly at concentrations up to 40 μM. UT represents untreated cells, DMSO indicates a solvent control, and proteasome inhibitor MG132 was used as a positive control. B, 4T1 Twist+ cells were treated with harmine at concentrations of 60, 120 and 180 μM. UT represents untreated cells, DMSO indicates a solvent control, and proteasome inhibitor MG132 and harmine at 500 μM were used as positive controls. All data summarize results from four independent experiments. Definitions of statistical significance are * p < .05, ** p < .01, *** p < .001, and **** p < .0001.

To investigate whether inhibition of breast cancer cell migration and invasion is due to cytotoxicity-related cell death induced by harmine, we performed a cell viability assay with a Cell Counting Kit-8 (CCK-8) (Materials and Methods) that detects the number of viable cells after harmine treatment. Viability of untreated BT549 Twist+ cells (UT) (negative control), BT549 Twist+ cells treated with DMSO (solvent control), or harmine at concentrations of 10, 20, and 40 μM, or MG132 proteasome inhibitor at a concentration of 10 μM (positive control) was analyzed. As shown in Fig 3A, DMSO and harmine did not cause significant changes in cell viability. We observed that even when the concentration of harmine was increased from 20μM to 40 μM, that is, twice as high as the concentration used in the wound healing and 3D invasion assays with BT549 cells, cell viability did not change significantly. As expected, MG132 at 10 μM caused significant inhibition of cell viability. This result suggests that harmine inhibits migration and invasion of BT549 cells independently of its cytotoxicity.

To verify the above result, we performed a similar assay with 4T1 cells. These cells were either untreated (UT) (negative control), treated with DMSO (solvent control), or harmine at the concentations of 60, 120, and 180 μM, or harmine at the concentration of 500 μM as a positive control due to its reported cytotoxicity at high concentration, or MG132 (positive control) for 24 hr. As shown in Fig 3B, DMSO did not cause any significant change in cell viability, and as expected, harmine at a high concentration and MG132 caused significant inhibition of cell proliferation. We found that at concentrations of 60, 120, and 180 μM, harmine causes a significant inhibition of cell proliferation. We also observed that the inhibitory effect of harmine increases significantly between concentrations of 60 μM and 120 μM, but does not change significantly from 120 to 180 μM. However, the moderate inhibition (P = 0.016) of 4T1 cell viability does not substantiate the significant inhibition in migration (P = 0.00026) and invasion (P = 0.00044) imposed by harmine by increasing the concentration from 60 to 120 μM (Figs 1 and 2), suggesting that harmine mainly inhibits the migration and invasion of 4T1 cells independently of its cytotoxicity, which is consistent with the result obtained from the BT549 cell assay.

### Harmine induces a dose-dependent degradation of Twist1

**Fig 4:**
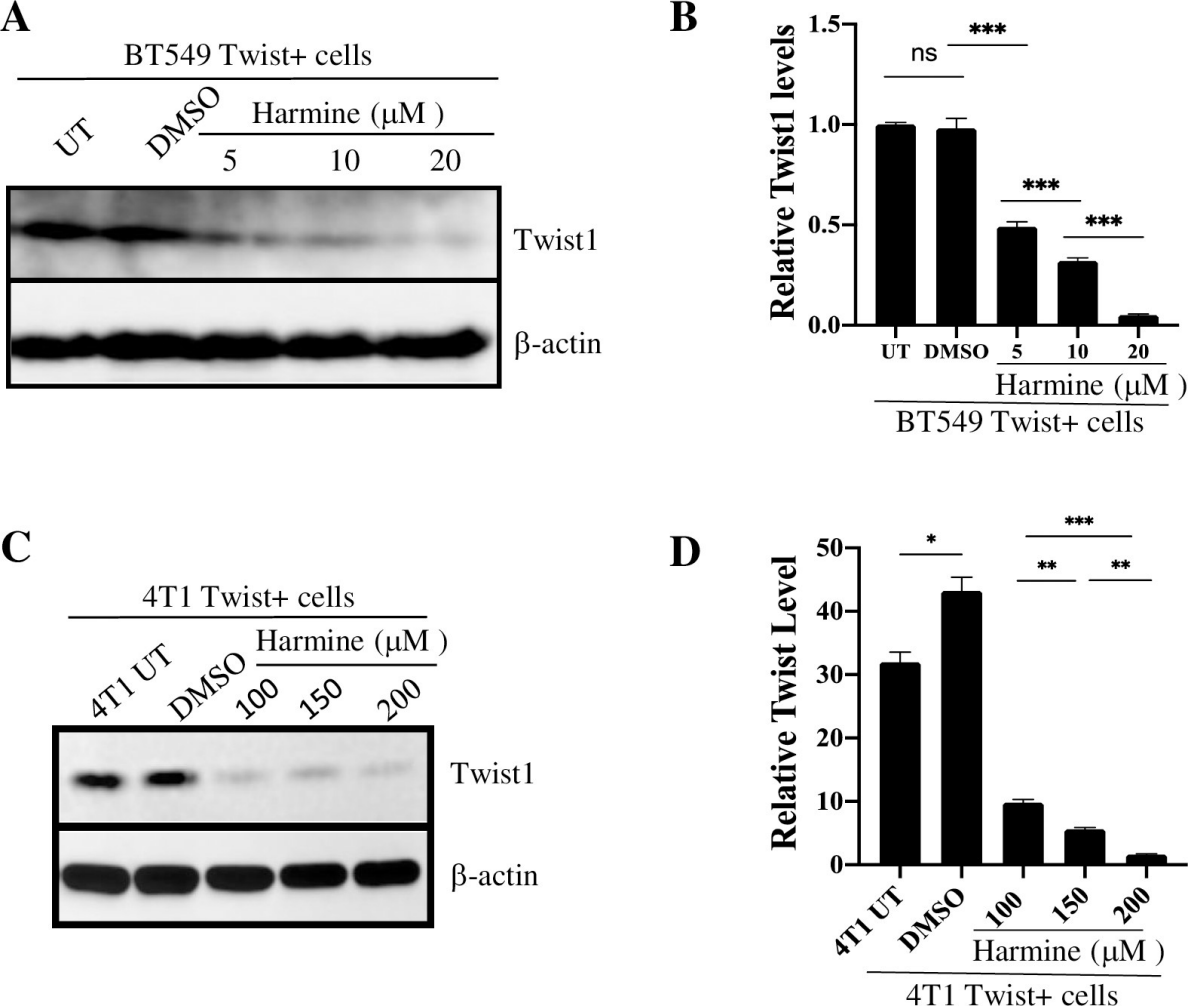
Harmine induces a dose-dependent degradation of Twist1 in breast cancer cells. A, The BT549 Twist+ cells were either untreated (UT), treated with DMSO, or treated with harmine at the concentrations indicated for 48 hr. Cell lysates were analyzed by blotting for Twist1 or β-actin (loading control). B, Quantitative analysis of result shown in A. C, The 4T1 Twist+ cells were either untreated (UT), treated with DMSO, or treated with harmine at the concentrations indicated for 48 hr. Cell lysates were analyzed by blotting for Twist1 or β-actin (loading control). D, Quantitative analysis of result shown in C. Definitions of statistical significance are * p < .05, ** p < .01, *** p < .001, and **** p < .0001.

Since Twist1 is a key regulator that promotes breast cancer cell invasion, and since harmine induces Twist1 degradation in NSCLC cells, we would like to know whether harmine-mediated inhibition of migration and invasion of breast cancer cells is due to harmine-induced degradation of Twist1 in these cells. For this purpose, BT549 Twist+ cells were either untreated (UT) (negative control), or treated with harmine at final concentrations of 5, 10, and 20 μM, or treated with DMSO (solvent control). After incubation at 37°C for 48 hrs, cells were harvested, lysed, and lysates analyzed by Western blotting to detect the level of Twist1. As shown in Fig 4A and 4B, we observed a harmine dose-dependent degradation of Twist1, where the level of Twist1 was reduced to ~10% when cells were treated with 20 μM harmine. Thus, for BT549 cells, we conclude that treatment with harmine at 20 μM is sufficient to cause significant reduction of Twist1 in BT549 cells.

To determine whether harmine also induces Twist1 degradation in 4T1 cells, we performed a similar experiment with these cells. Consistent with our discovery using wound healing and 3D invasion assays, we initially treated the 4T1 Twist+ cells with 20 μM of harmine for 48 hr but did not observe any significant decrease in Twist1 level. We then treated the cells with harmine at concentrations of 100, 150, and 200 μM, with untreated cells (4T1 UT) or DMSO-treated cells as negative and solvent controls, respectively. We found that harmine causes a significant degradation of Twist1 in all cells, and this degradation is dose-dependent (Fig 4C and 4D). Thus, these results demonstrate that harmine induces a dose-dependent degradation of Twist1 in breast cancer cells, which is probably the cause of inhibition of cellular migration and invasion.

### Harmine-induced Twist1 degradation is proteasome-dependent, and degradation of Twist1 is facilitated by cycloheximide

**Fig 5:**
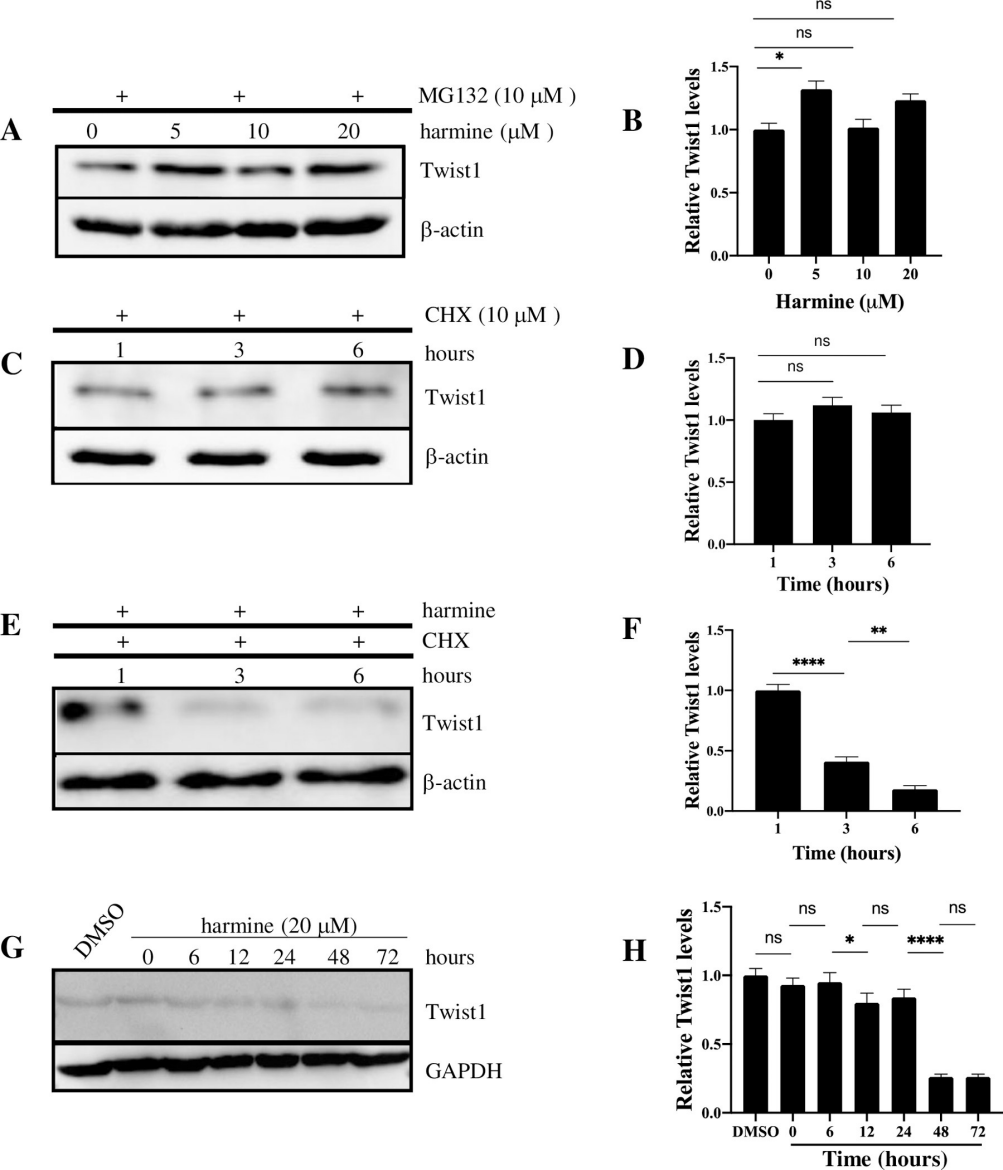
Harmine-induced Twist1 degradation in breast cancer cells is proteasome-dependent. A, BT549 Twist+ cells were treated with harmine at the concentrations indicated for 48 hr in the presence of 10 μM MG132. Cell lysates were analyzed by blotting for Twist1 or β-actin (loading control). B, Quantitative representation of result shown in A. C, BT549 Twist+ cells were treated with cycloheximide (CHX) at a concentration of 10 μM for 48 hr. Cell lysates were analyzed by blotting for Twist1 or β-actin (loading control). D, Quantitative representation of result shown in C. E, BT549 Twist+ cells were treated with both cycloheximide (10 μM) and harmine (20 μM) for 48 hr. Cell lysates were analyzed by blotting for Twist1 or β-actin (loading control). F, Quantitative representation of result shown in E. G, BT549 Twist+ cells were treated with either DMSO (solvent control) or harmine at a concentration of 20 μM and cells were collected at timepoints indicated. Cells treated with DMSO were collected at 72 hr. Cell lysates were analyzed by blotting for Twist1 or GAPDH (loading control). H, Quantitative representation of result shown in E. Definitions of statistical significance are * p < .05, ** p < .01, *** p < .001, and **** p < .0001.

Since harmine probably inhibits cell migration and invasion by inducing Twist1 degradation, we asked whether degradation of Twist1 is proteasome-dependent. Proteasome inhibitor MG132 was added to BT549 Twist+ cells to a final concentration of 10 μM with harmine at concentrations of 0 (DMSO control), 5, 10, and 20 μM. After 48 hr, cells were harvested and lysed, and cell lysates were analyzed by Western blotting. As shown in Fig 5A and 5B, the level of Twist1 was largely unchanged with harmine treatment at all concentrations, suggesting that degradation of Twist1 is proteasome-mediated. In order to better understand the turnover time of Twist1 *in vivo*, we compared the degradation rate of Twist1 after treatment with either cycloheximide + harmine or cycloheximide alone. As shown in Fig 5C and 5D, with cycloheximide, which inhibits protein synthesis alone, the Twist1 level was stable for at least 6 hrs. This suggests that Twist1 has a relatively long half-life. On the other hand, when the cells were treated with both cycloheximide and harmine (Fig 5E and 5F), Twist1 degradation was detected at as early as 3 hr post treatment, suggesting that harmine can rapidly induce Twist1 degradation *in vivo*. To further elucidate the time course of Twist1 degradation, we treated BT549 Twist+ cells with DMSO or harmine at a concentration of 20 μM and collected samples at 0, 6, 12, 24, 48, and 72 hr post treatment. Cell lysates were subjected to blot for Twist1. As shown in Fig 5G and 5H, the Twist1 level was relatively stable until 24 hr post treatment, but by 48 hr after treatment was reduced to ~20% of that at time 0. Further treatment to 72 hrs did not reduce Twist1 level further. This is consistent with the results shown in Figs 1 and 2 with BT549 cells where differences in migration and invasion were shown after 48 hr.

In summary, these results suggest that harmine-induced Twist1 degradation is proteasome-dependent. Without *de novo* synthesis of Twist1, it could be degraded quickly. However, without the inhibition of protein synthesis, it took up to 48 hours to observe significant degradation of Twist1 in the presence of harmine in BT549 cells.

### Harmine does not regulate either expression of Twist1 or stability of Twist1 mRNA

**Fig 6:**
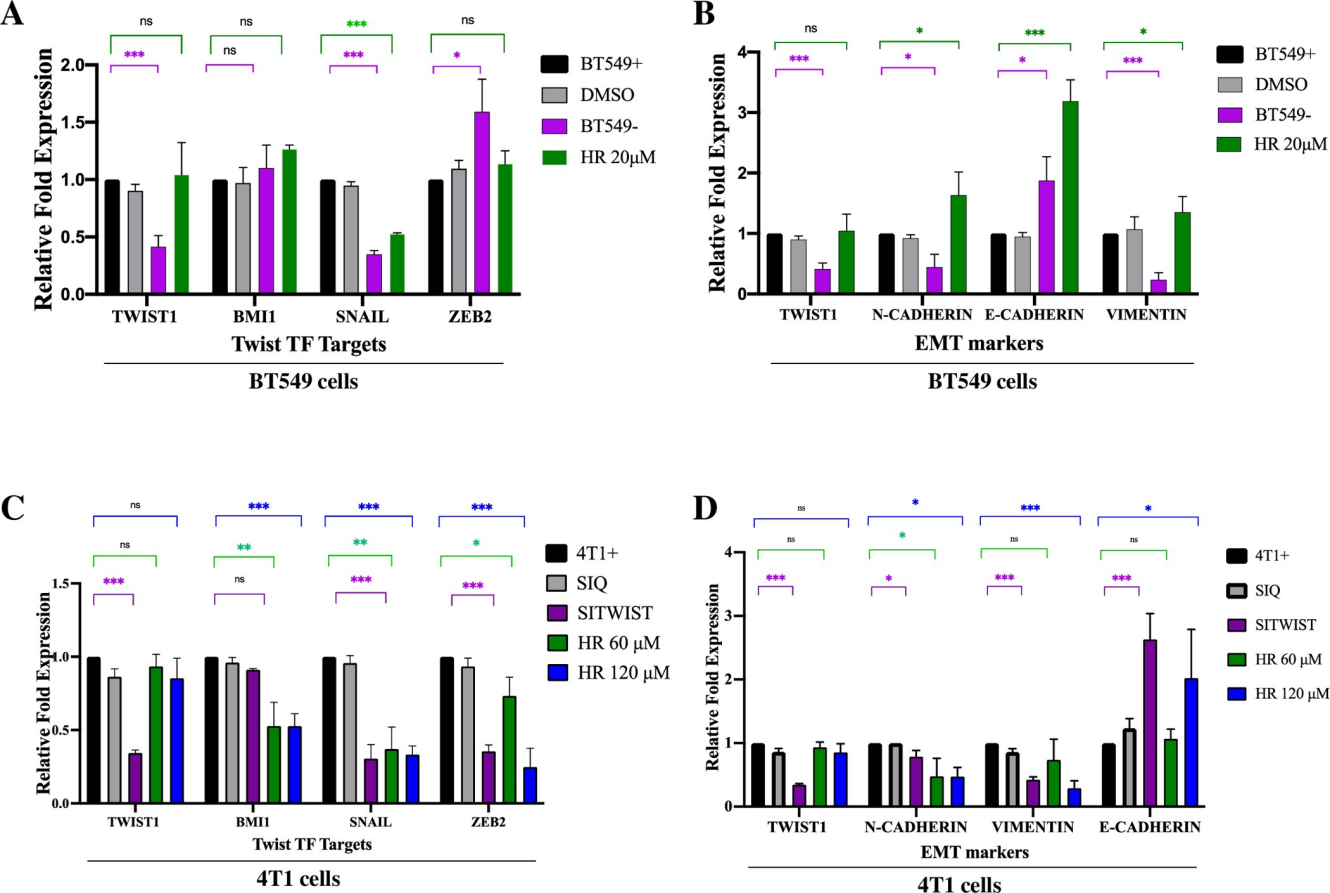
Harmine treatment does not affect the level of Twist1 transcripts in breast cancer cells. A, Analysis of the expression of Twist1 and its target genes in BT549 cells. BT549 Twist+ cells were either untreated (BT549+), treated with DMSO (DMSO) or harmine (HR 20μM) for 48 hr, and the levels of Twist1 mRNA were determined by RT-qPCR (see Materials and Methods). BT549 Twist- cells (BT549-) were included as a negative control. The mRNA levels of Twist1 and its target genes Bmi1 (BMI1), Snail (SNAIL) and ZEB2 (ZEB2) were normalized against that of β-actin, tubulin α 1a and GAPDH. B, The expressions of EMT markers N-Cadherin (N-CADHERIN), E-Cadherin (E-CADHERIN) and Vimentin (VIMENTIN) were analyzed with the same samples as in A. C, Analysis of the expression of Twist1 and its target genes in 4T1 cells. The 4T1 Twist+ cells were either untreated (4T1+), transfected with scrambled control siRNA (SIQ), transfected with Twist1 siRNA (SITWIST) (negative control), treated with 60 μM harmine (HR 60 μM) or treated with 120 μM harmine (HR 120 μM). mRNA levels for Twist1 and its target genes Bmi1 (BMI1), Snail (SNAIL) and ZEB2 (ZEB2) were measured by RT-qPCR. All results were normalized against the mRNA levels of β-actin, tubulin α 1a and GAPDH. D, The expressions of EMT markers N-Cadherin (N-CADHERIN), E-Cadherin (E-CADHERIN) and Vimentin (VIMENTIN) were analyzed with the same samples as in C. All data were pooled from three independent experiments. Definitions of statistical significance are * p < .05, ** p < .01, *** p < .001, and **** p < .0001.

To find out whether harmine could also reduce Twist1 levels by inhibiting transcription of *Twist1* or destabilizing Twist1 mRNA, we performed RT-qPCR to analyze the expression levels of Twist1 and its representative downstream targets. Untreated BT549 Twist+ cells, treated with DMSO or 20 μM harmine, together with BT549 Twist- cells were harvested and total RNA was extracted. After RT-qPCR (see Materials and Methods), the mRNA levels of Twist1, Twist1 transcription targets Bmi1 (BMI-1), Snail (SNAIL) and ZEB2 (ZEB2) were normalized against mRNA levels of β-actin, tubulin α 1a, and GAPDH and to untreated BT549 Twist+ cells. As shown in Fig 6A, treatment with DMSO or harmine did not cause any significant change in the level of Twist1 mRNA, suggesting that harmine-caused Twist1 reduction does not involve regulation of Twist1 mRNA. As expected, BT549 Twist- cells have a significantly lower level of Twist1 mRNA. We observed that in BT549- cells, expression of Bmi1 was largely unchanged, expression of Snail was significantly reduced, and expression of ZEB2 increased, suggesting that Twist1 may not be the only transcriptional regulator of these genes. We also observed that harmine causes a significant reduction of Snail expression, but no significant change in the expression of Bmi1 and ZEB2, suggesting that inducing the degradation of Twist1 alone is not sufficient to reduce the expression of Bmi1 and ZEB2. When analyzing the expression of EMT related genes (Fig 6B), we found that harmine causes a significant increase in the expression of epithelial factor E-Cadherin, which is expected, but that expression of N-Cadherin and Vimentin also increased. This suggests that harmine might have affected the expression of these genes by an as yet unknown mechanism in BT549 cells.

To elucidate the effect of harmine on the expression of Twist1 and its target genes in 4T1 cells, we extracted total RNAs from untreated 4T1 Twist+ cells, 4T1 Twist+ cells transfected with scrambled siRNA or siRNA targeting Twist1, or treated with harmine at the concentrations of 60 μM, or 120 μM. Similarly to the experiment with BT549 cells, we examined mRNA levels of Bmi1, Snail, and ZEB2. All results were normalized with mRNA levels of β-actin, tubulin α 1a and GAPDH and normalized to the untreated 4T1 cells (negative control cells). As shown in Fig 6C, while the Twist1 mRNA level did not change significantly after harmine treatment, as expected, mRNA expression of its targets, Bmi1, Snail, and ZEB2, were significantly reduced due to the inhibition of Twist1 by harmine. As expected, the Twist1 mRNA level was significantly reduced in 4T1 Twist- cells, as were mRNA levels of Snail and ZEB2. However, Bmi1 expression level remained largely unchanged in these cells, suggesting that other mechanisms may contribute to the regulation of Bmi1 expression.

Our results also showed a significant reduction in the expression of Twist1-regulated EMT markers such as N-Cadherin and Vimentin after treatment with 120 μM harmine. On the other hand, E-Cadherin showed a significant increase in mRNA level (Fig 6D). In summary, these results strongly suggest that the reduction of Twist1 protein level by harmine does not affect Twist1 mRNA level. In addition, the inhibition of Twist1 by harmine also suppresses the expression of its downstream transcriptional targets, including Snail, ZEB2, and N-Cadherin and Vimentin in 4T1 cells. However, the effect of harmine on the expression of Twist1 transcription targets and related EMT markers is complex and needs further investigation.

## Discussion

In this study, we show that a beta-carboline alkaloid, harmine, is capable of inhibiting breast cancer cell migration and invasion, which are the initial steps towards metastasis. This is accomplished by inducing proteasome-mediated degradation of Twist1 in a dose dependent manner. Twist1 is overexpressed in a variety of cancers and is a well-documented master regulator of breast cancer metastasis. It promotes EMT and cell invasion by activating several target genes, including Bmi1, ZEB2, and Snail, that increase cellular de-differentiation and cell mobility. Because of the importance of Twist1 in metastasis, we also examined the expression of EMT markers E-Cadherin, N-Cadherin, and Vimentin in harmine-treated cancer cells and demonstrated that reduced Twist1 affects the Twist1-regulated downstream targets involved in EMT. Thus, harmine may be an attractive therapeutic target.

The use of siRNA for cancer therapy is of considerable interest due to its gene silencing properties. However, while promising, the application of siRNA-based therapy is limited by several obstacles, for example susceptibility to enzymatic degradation, difficulty of delivery to target tissues, off-target effects, etc. By using a modified PAMAM dendrimer, we knocked down Twist1, reduced the expression of EMT-related target genes, and altered the phenotypic characteristics associated with cancer cell migration and invasion. While this siRNA approach could serve as a valuable tool and adjuvant therapy to reduce migration/invasion, chemoresistance, and anti-apoptotic tendencies associated with aggressive tumors, it may not be used as a sole means of treatment for metastatic breast cancer. The observation that harmine induces Twist1 degradation and therefore inhibits cancer cell invasion provides a possible alternative approach. In this study, we found that harmine can suppress the expression of both Bmi1 and Snail, the downstream targets of Twist1, but Twist1 siRNA only suppresses the expression of Snail and ZEB2, but not Bmi1, within 48 hrs. This could be due to a number of possibilities. For example, expression of Bmi1 may require less Twist1 to activate as the siRNA did not completely block Twist1 expression, or Bmi1 mRNA has a longer half-life, or a combination of both. Harmine, however, induces Twist1 degradation within three hours, resulting in downstream changes to EMT markers, such as increased levels of E-Cadherin and decreased levels of Vimentin and N-Cadherin. These results suggest that targeting Twist1 protein could be an effective approach either alone or as a supplement to other measures such as siRNA in metastatic breast cancer therapy.

How does harmine induce proteasome-dependent Twist1 degradation? First, we need to understand how Twist1 stability is regulated. In vertebrates, Twist1 forms a heterodimer with an E protein to gain transactivation capability. In NSCLC cells, both E12 and E47 can dimerize with Twist1, they reciprocally stabilize each other, and heterodimers significantly increase transcription of Twist1 target genes compared to the Twist1-Twist1 homodimer or Twist1 monomer [28]. Furthermore, biochemical analyses show that the WR domain, the C-terminal 20 residues of Twist1, dimerizes to mediate tetramer formation, which is functionally required for Twist1 to induce EMT [30]. Previously, we showed that the WR domain mediates a binding interaction between Twist1 and the NF-κB subunit RELA to transcriptionally upregulate the inflammatory cytokine interleukin 8 (IL-8) [29]. It was also reported that the WR domain binds the C-terminus of the tumor suppressor p53, leading to p53 degradation [31].

Yochum et al. showed that harmine preferentially induces degradation of the Twist1-E2A heterodimer rather than the Twist1-Twist1 homodimer and that degradation of the heterodimer is required for the inhibitory effects of harmine [28]. In another study, Lander et al. showed that the WR domain is required for Twist ubiquitination and that deletion of the WR domain stabilizes Twist. However, the WR domain does not contain the lysine residues required for an ubiquitin link. The authors further showed that the WR domain interacts with F-box protein Partner of paired (Ppa), which serves as the substrate recognition component of a Skp–Cullin–F-box E3 ubiquitin ligase. It is therefore possible that harmine promotes or stabilizes the interaction between the WR domain and Ppa to facilitate the degradation of Twist1 and/or E2a proteins. A recent study showed that PKCα phosphorylates Twist1 at serine 144 located in the loop region, which precludes Twist1 ubiquitination and stabilizes Twist1 [32]. Thus, it is also possible that harmine somehow prevents phosphorylation and therefore destabilizes Twist1.

In addition to inducing Twist1 degradation, harmine can also inhibit breast cancer cell proliferation and migration by inhibiting the expression of TAZ [27]. TAZ is a downstream effector of the Hippo pathway that plays an important role in mammalian development [33]. TAZ is reported to be an oncogene in certain types of human cancers, including breast cancer, and promotes cancer cell migration and invasion [34]. The authors claimed that by inhibiting the expression of TAZ, harmine executed its anticancer activity. Thus, harmine may inhibit breast cancer cell migration and invasion through multiple mechanisms.

In conclusion, our study shows that harmine induces Twist1 degradation in a dose dependent manner in breast cancer cells and inhibits their migration and invasion. This degradation is mediated by proteasomes and is unlikely to involve regulation of Twist1 gene expression or mRNA stability, but does affect expression of Twist1 target genes involved with EMT, particularly in 4T1 cells. The exact mechanism by which harmine promotes Twist1 degradation is not clear, but future studies are underway to understand its mechanism and thus help develop new therapeutic approaches to treat metastatic breast cancer.

## Supporting information

S1 Fig (TIF)

S1 Raw images (PDF)
