# Supplementary figures and images for: Harmine inhibits breast cancer cell migration and invasion by inducing the degradation of Twist1
Source: PLoS One. 2021 Feb 24;16(2):e0247652. doi: 10.1371/journal.pone.0247652 (PMC7904211; doi:10.1371/journal.pone.0247652)

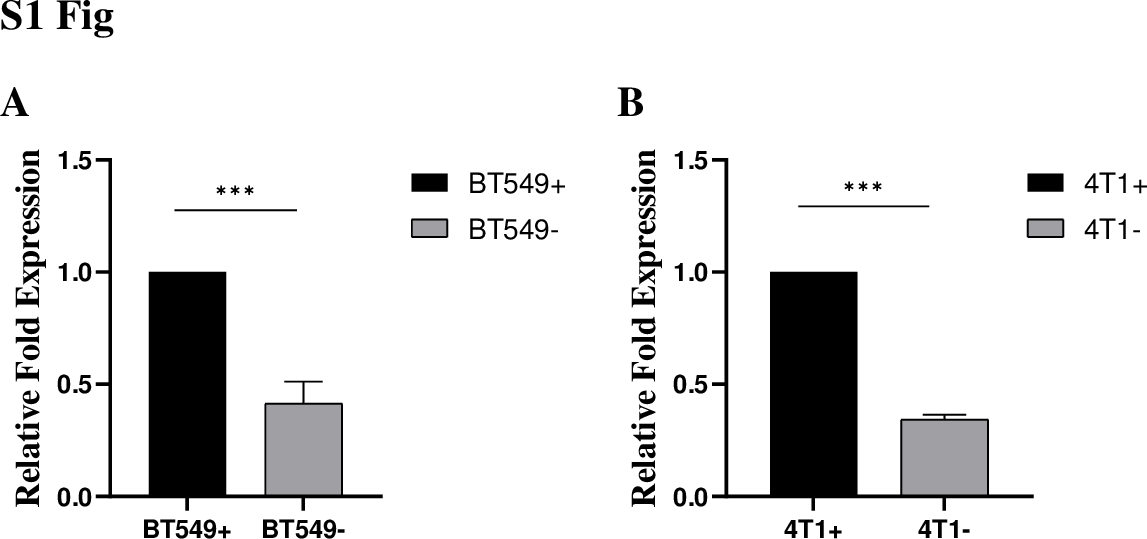

Supplement: S1 Fig — (TIF) [file pone.0247652.s001.tif]
