# Supplementary material for: Harmine inhibits breast cancer cell migration and invasion by inducing the degradation of Twist1
Source: PLoS One. 2021 Feb 24;16(2):e0247652. doi: 10.1371/journal.pone.0247652 (PMC7904211; doi:10.1371/journal.pone.0247652)
Supplement: S1 Raw images — (PDF) [file pone.0247652.s002.pdf]

Fig 4A raw images

Fig 4A Twist1 blot

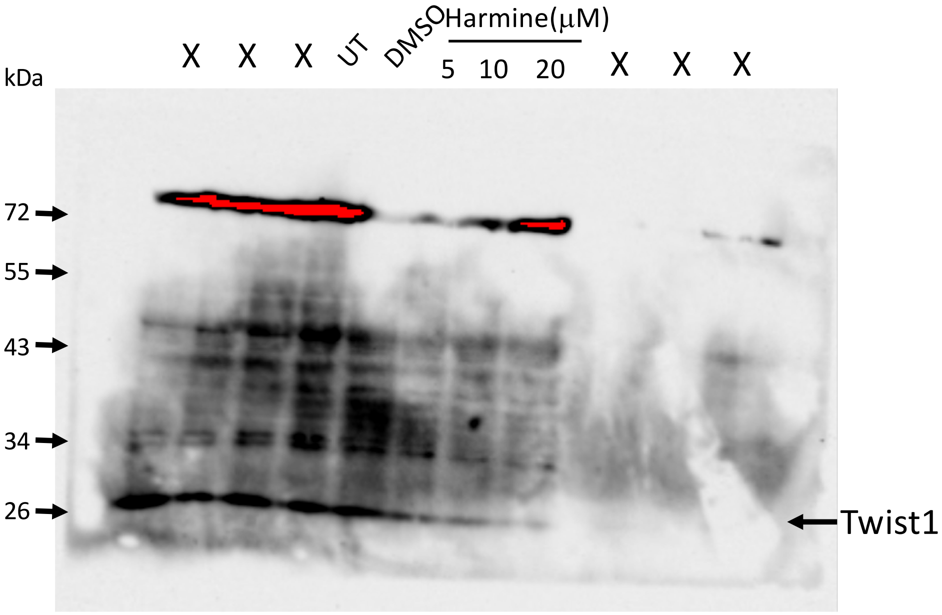

Fig 4A loading control blot

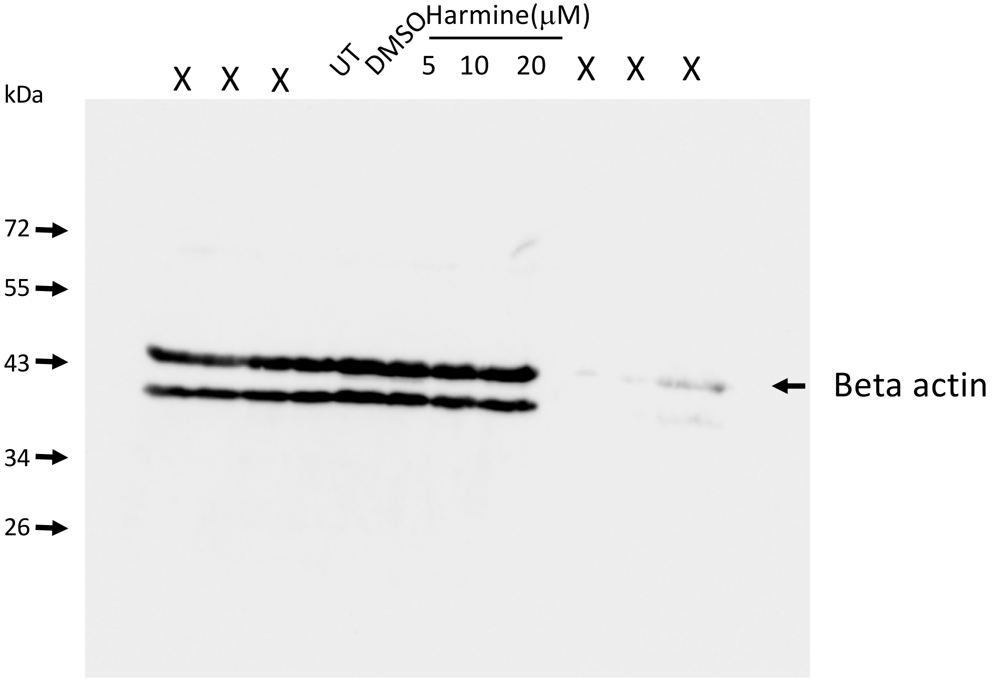

The images were captured with Bio-Rad ChemiDoc imager

Fig4C raw images

Fig 4C-Twist1 blot

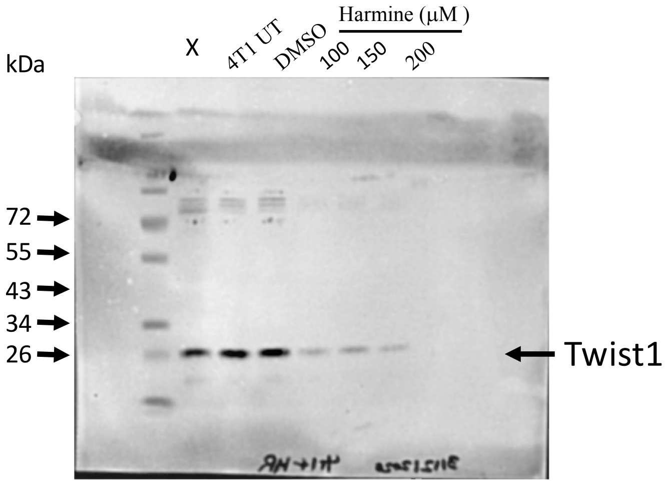

Fig 4C-loading control blot

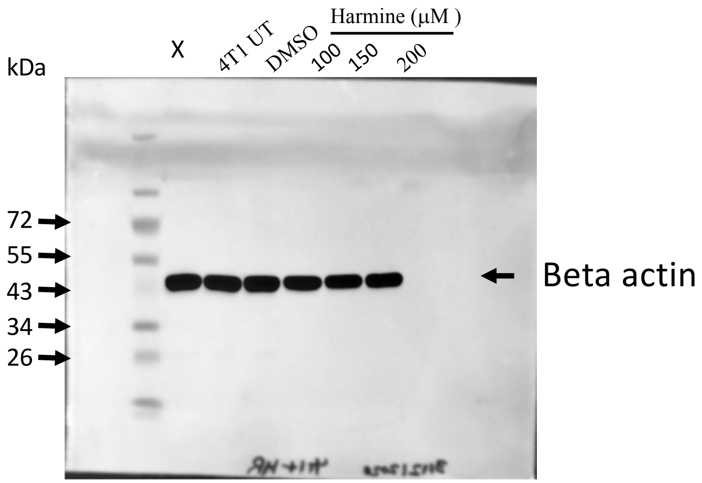

The images were captured with Bio-Rad ChemiDoc imager

Fig 5A raw images

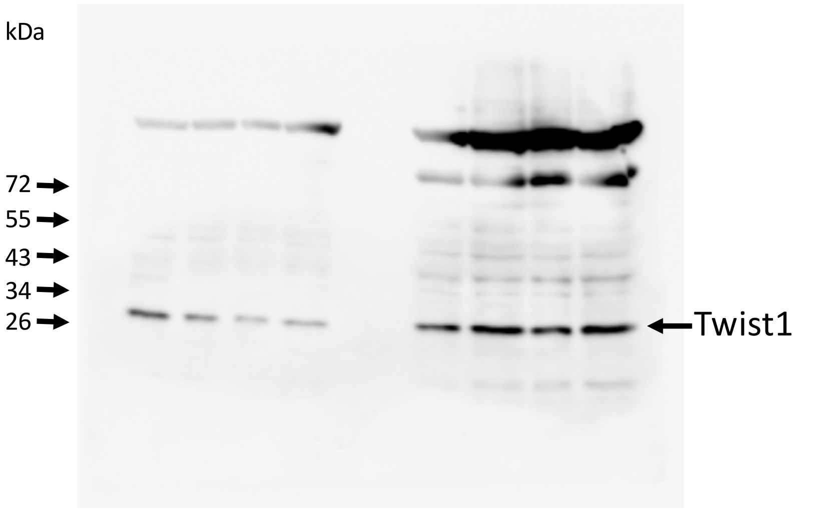

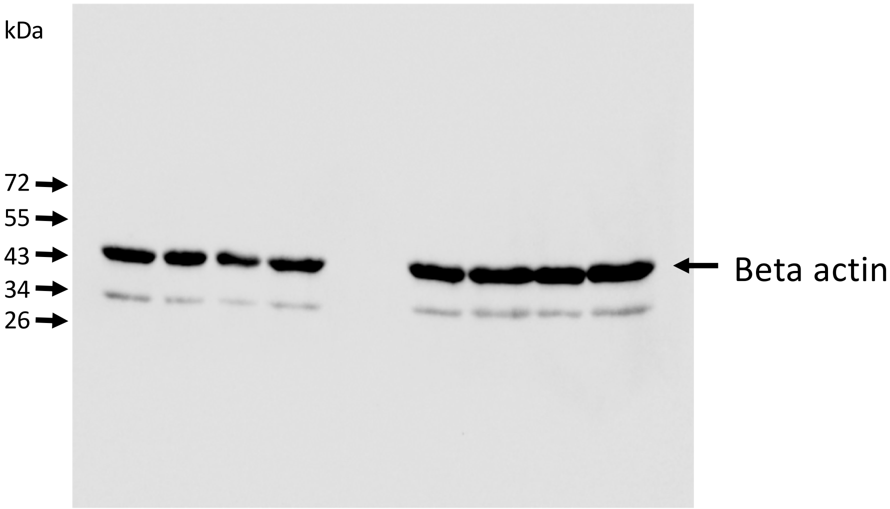

The images were captured with Bio-Rad ChemiDoc imager

Fig 5C raw images

Fig 5C Twist1 blot

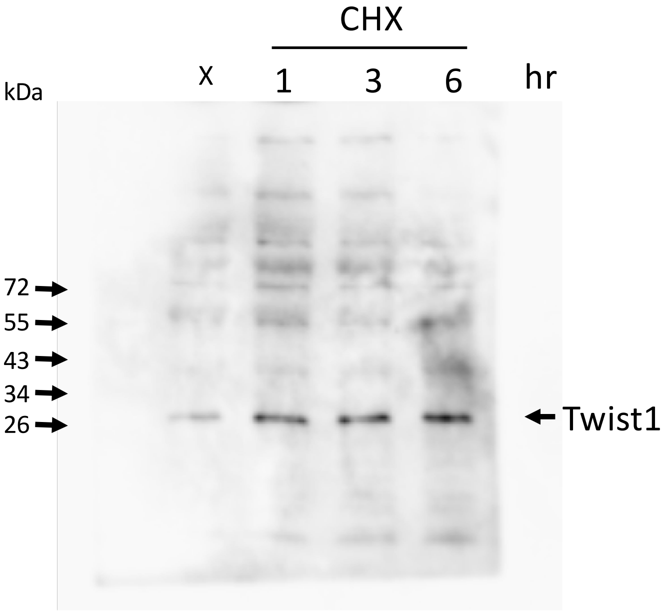

Fig 5C loading control blot

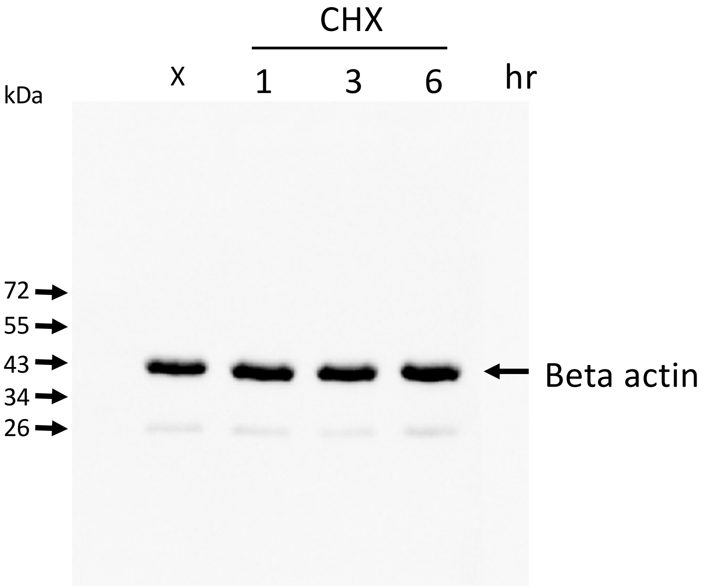

The images were captured with Bio-Rad ChemiDoc imager

Fig 5E raw images

Fig 5E Twist1 blot

					+	+	+						Harmine
					+	+	+						CHX
x	x	x	x	x	1	3	6	x	x	x	x	x	hr

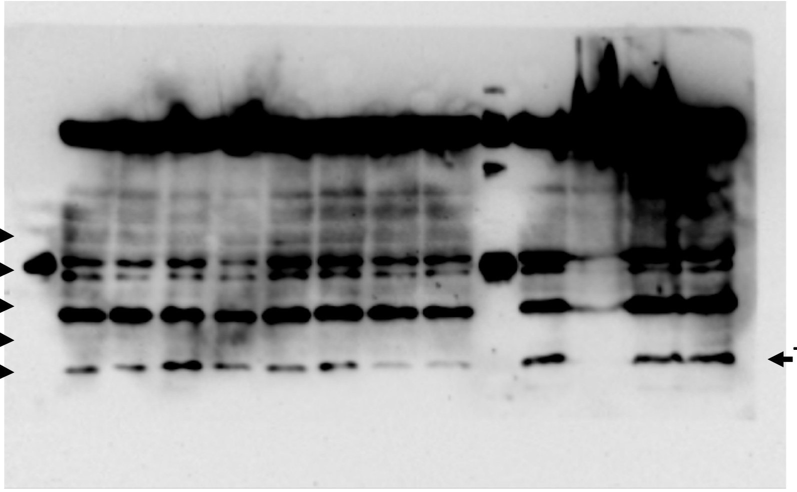

Fig 5E loading control blot

					+	+	+						Harmine
					+	+	+						CHX
x	x	x	x	x	1	3	6	x	x	x	x	x	hr

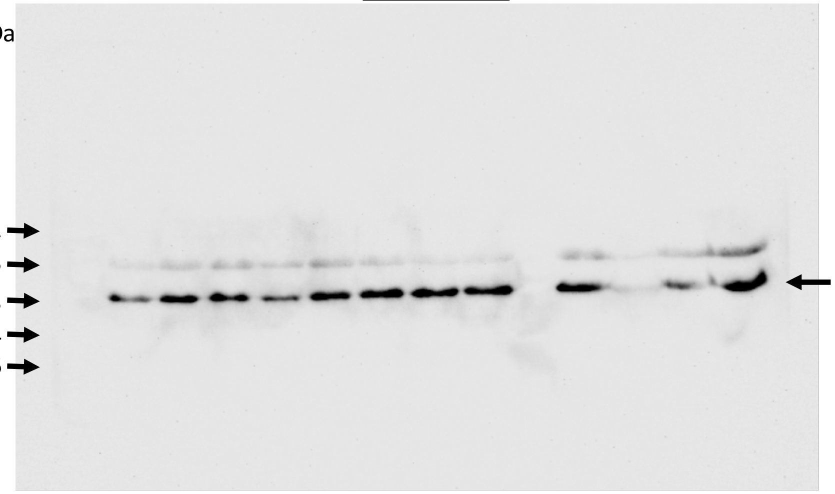

The images were captured with Bio-Rad ChemiDoc imager

Fig 5G raw images

Fig 5G Twist1 blot

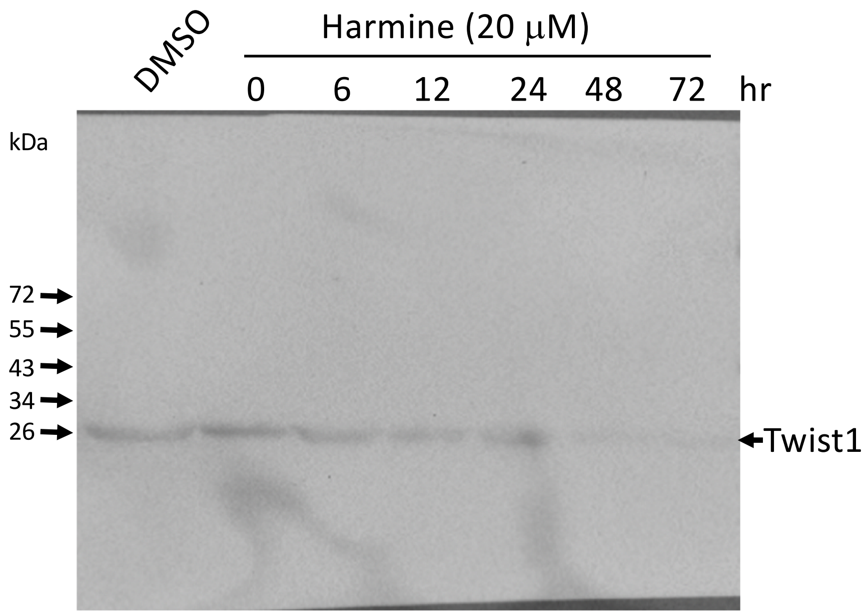

Fig 5G loading control blot

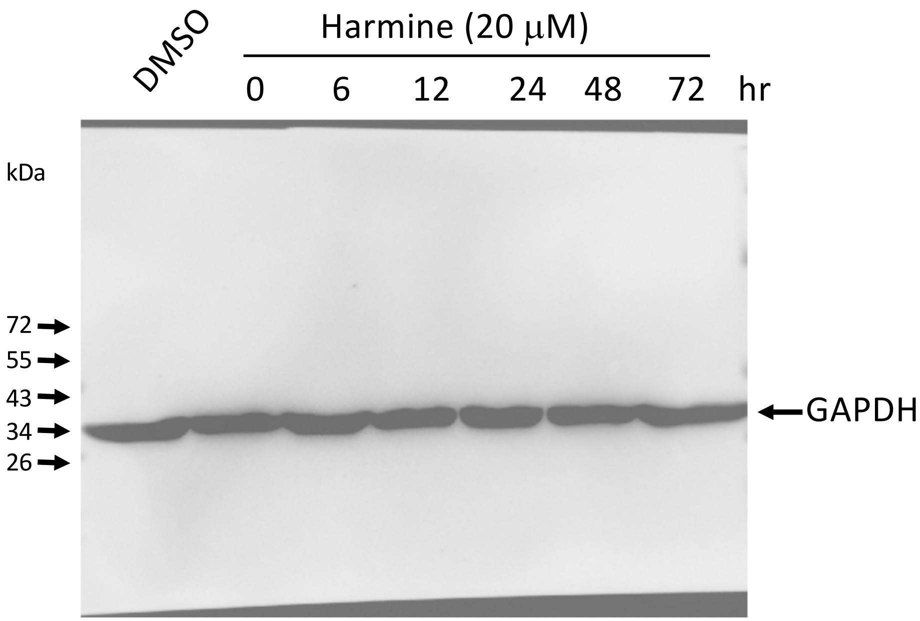

The images were captured with Bio-Rad ChemiDoc imager
